# Value of serum free prostate-specific antigen density in the diagnosis of prostate cancer

**DOI:** 10.1007/s11845-023-03448-w

**Published:** 2023-07-06

**Authors:** Bing-Zi Zou, Hong Wen, Huan-Jia Luo, Wan-Chao Luo, Qi-Tong Xie, Meng-Ting Zhou

**Affiliations:** 1 Department of Medical Ultrasonics, Huizhou Central People’s Hospital, Huizhou, Guangdong People’s Republic of China; 2 Department of Urology, Huizhou Central People’s Hospital, Huizhou, Guangdong People’s Republic of China

**Keywords:** Concordance, Diagnosis, Free prostate-specific antigen density, Prostate cancer

## Abstract

**Purpose:**

To investigate the value of serum free prostate-specific antigen density (fPSAD) in the diagnosis of prostate cancer (PCa).

**Methods:**

The data of 558 patients who underwent transrectal ultrasound-guided prostate biopsy were retrospectively analyzed. According to the pathological results, the patients were divided into a PCa group and a benign prostatic hyperplasia (BPH) group. Receiver operating characteristic curves were plotted, based on which the sensitivity, specificity, Youden index, concordance, and kappa values of free prostate-specific antigen (fPSA), the free-to-total f/tPSA, prostate-specific antigen density (PSAD), the free-to-total (f/t)/PSAD ratio, and fPSAD were compared. The patients were divided into three groups by PSA levels (PSA < 4 ng/mL, PSA = 4–10 ng/mL, and PSA > 10 ng/mL), into three groups by age (age < 60 year, age = 60–80y, and age > 80 years), and into two groups by prostate volume (PV) (PV ≤ 80 mL and PV > 80 mL) to compare the sensitivity, specificity, and concordance of indicators.

**Results:**

tPSA, PSAD, (f/t)/PSAD, and fPSAD had high accuracy in predicting PCa with AUC values of 0.820, 0.900, 0.846, and 0.867. fPSAD showed lower diagnostic sensitivity but significantly higher specificity and concordance for PCa than tPSA, f/tPSA, (f/t)/PSAD, or PSAD. Thus, fPSAD had the highest accuracy in the diagnosis of PCa. In the groups with different PSA, age, and PV stratification, the concordance of fPSAD was significantly higher (88.61%, 90.74%, and 90.38%) than that of other indicators.

**Conclusion:**

With the optimal cutoff value of 0.062, fPSAD has a higher diagnostic value for PCa than tPSA, f/tPSA, (f/t)/PSAD, and PSAD, and can well predict the risk of PCa, significantly improve the clinical diagnostic rate of PCa, and reduce unnecessary biopsy.

## Introduction

Prostate cancer (PCa) is one of the most common cancers in men and has the second-highest incidence rate among cancers globally [1]. In recent years, the annual incidence rate of PCa has been increasing in China [2]. Prostate-specific antigen (PSA) is an important marker for PCa screening, with high tissue specificity. However, the PSA level is also abnormally elevated in many benign prostate diseases, leading to high missed diagnosis and misdiagnosis rates [3–6]. Prostate biopsy is the gold standard for PCa diagnosis. Transrectal ultrasound (TRUS)-guided biopsy is the most critical diagnostic means for PCa. However, it may lead to complications, such as hematuria, hemospermia, rectal bleeding, and even sepsis in severe cases [7]. Given the incidence of these complications, patients requiring biopsy must be screened to avoid unnecessary biopsy.

Many studies have focused on PSA and PSA-derived indicators, such as the free/total PSA (f/tPSA) ratio and PSA density (PSAD), which both have higher diagnostic accuracy than PSA. Moreover, as found in a previous study among people with a PSA level of 4–10 ng/mL, the index (f/t)/PSAD has higher specificity than f/tPSA or PSAD alone [8].

Lin et al. [9] reported the high accuracy of free PSAD (fPSAD) in predicting prostate biopsy results in 2017. Based on this study, we further assessed the diagnostic accuracy of fPSAD among 558 Chinese patients, thereby providing further support for the clinical diagnosis of PCa.

## Materials and methods

### Baseline data

The data of patients who underwent systematic prostate biopsy in Huizhou Municipal Central Hospital from January 2014 to July 2021 were retrospectively collected. According to the pathological results of prostate biopsy, the patients were divided into a PCa group and a benign prostatic hyperplasia (BPH) group. The prostate biopsy followed the guidelines of the Chinese Urology Association: (1) suspicious prostate nodules were found by digital rectal examination (DRE), (2) suspicious lesions were detected by TRUS or magnetic resonance imaging (MRI), (3) PSA > 10 ng/mL, regardless of the value of f/tPSA and PSAD, and (4) PSA = 4–10 ng/mL, abnormal f/tPSA or abnormal PSAD value. PCa or BPH was diagnosed by biopsy. Inclusion criteria were as follows: (1) the patient had his first diagnosis of PCa, (2) he was undergoing prostate biopsy (systematic + targeted) for the first time, and (3) he had not undergone any treatment before the biopsy. Exclusion criteria: (1) history of PCa, PCa surgery, or drug therapy, (2) urinary tract infection or obstruction, (3) diagnosis of prostatitis, or (4) prostatic massage, DRE, TRUS, or cystoscopy that might affect the serum PSA level within 2 weeks before the PSA test.

#### TRUS

Complete scanning of the prostate was conducted on the transverse section and sagittal section with the TRUS probe lightly touching the prostate. The transverse diameter (left and right diameter) of the prostate was measured on the maximum transverse section, and craniocaudal diameter (up and down diameter) and anteroposterior diameter (front and back diameter) of the prostate were measured on the median sagittal section of the prostate passing through the internal orifice of the urethra. The measured dimensions of the prostate were recorded as transverse diameter × anteroposterior diameter × craniocaudal diameter. To objectively measure prostate volume, it was necessary to avoid prostate deformation by forced extrusion and to ensure that the measurement was performed on the maximum transverse section and median sagittal section of the prostate.

### Prostate biopsy

All patients were examined with a Philips EPIQ7 Ultrasound System and rectal convex array probes, and a BioPince™ Full Core Biopsy Instrument (manufacturer: Argon Medical Devices, Inc.) was used as the biopsy needle. Routine bowel preparation and bladder emptying were done before the biopsy. The patient was placed in a lateral decubitus or lithotomy position, and the US probe wrapped with a condom was slowly inserted from the anus. The probe was adjusted to display the image of the prostate, and a systematic prostate biopsy with a 12 + X needle was performed under the guidance of ultrasound. The biopsy sites were at the inner and outer sides of the left and right prostatic apex, the inner and outer sides of the prostatic body, and the inner and outer sides of the prostatic base. Another 1–3 sites could be selected based on the size of the suspicious area of the prostate detected by MR or US. Finally, the harvested specimen was marked and fixed in strips.

### Statistical analysis

IBM SPSS Statistics 22 software was used for statistical analysis. Measurement data are expressed as ($\overline{x }$ ± s). The independent-sample *t*-test was performed to compare means between two groups in the case of normally distributed data. Otherwise, the nonparametric test (Mann–Whitney *U* test) was performed. The paired chi-squared (*χ*^2^) test was performed to compare proportions between the two groups. *p* < 0.05 was considered statistically significant. Receiver operating characteristic (ROC) curves were plotted using GraphPad Prism 9, and the area under the ROC curve (AUC) was calculated with SPSS software. The pathological result of the prostate biopsy was the gold standard for the diagnosis of PCa.

The normal values of tPSA f/tPSA and PSAD were defined as < 4 ng/mL, > 0.16, and < 0.15, respectively, per the *2019 Chinese Guidelines for the Diagnosis and Treatment of Urological Diseases*. tPSA < 4 ng/mL, f/tPSA > 0.16, and PSAD < 0.15 were defined as negative predictions, while tPSA > 4 ng/mL, f/tPSA < 0.16, and PSAD > 0.15 as positive predictions. Based on the level of fPSAD and the gold standard, the ROC curve of fPSAD for predicting PCa was plotted, and the AUC was calculated. The Youden index values of the newly introduced parameters (f/t)/PSAD and fPSAD were calculated according to sensitivity and specificity. Moreover, the optimal cutoff points of the two for diagnosing PCa were selected given the maximum Youden index from the coordinate points of the ROC curve, and the negative and positive prediction results were defined.

Consistency evaluation of diagnostic results: the kappa value and *p* value of tPSA, f/tPSA, PSAD, (f/t)/PSAD, and fPSAD in predicting PCa were calculated by the paired chi-squared test, and their consistency with the pathological results was evaluated.

Then, the sensitivity, specificity, Youden index, and concordance of tPSA, f/tPSA, PSAD, (f/t)/PSAD, and fPSAD were calculated using the following formulas, and the value of these indicators for predicting PCa was compared. Calculation formula: PV = 0.52 × (transverse diameter) × (anteroposterior diameter) × (craniocaudal diameter); sensitivity = true positive/(true positive + false negative); specificity = true negative/(false positive + true negative); Youden index = (sensitivity + specificity) − 1; concordance = (true positive + true negative)/(true positive + false positive + true negative + false negative).

## Results

### Characteristics of included patients

A total of 558 patients were included, including 249 patients (45%) with PCa and 309 patients (55%) with BPH. The nonnormally distributed data were compared by the nonparametric test (Mann–Whitney *U* test) between the PCa and BPH groups. As shown in Table 1, the PCa group and the BPH group had no significant difference in f/tPSA but had significant differences in age, PV, tPSA, PSAD, (f/t)/PSAD, and fPSAD (*p* < 0.001).

**Table 1:** Characteristics of included patients

	*n*	Age	PV	tPSA	f/tPSA	PSAD	(f/t)/PSAD	fPSAD
PCa	249	71.42 ± 8.03	43.84 ± 25.59	72.46 ± 67.59	0.20 ± 0.18	1.86 ± 2.01	0.54 ± 2.45	0.41 ± 0.61
BPH	309	67.14 ± 8.79	66.20 ± 40.27	17.72 ± 20.23	0.18 ± 0.25	0.33 ± 0.71	1.44 ± 2.75	0.05 ± 0.13
*p*	/	0.000	0.000	0.000	0.648	0.000	0.000	0.000

*p* ≤ 0.05 was considered to be statistically significant

### Diagnostic performance of tPSA, f/tPSA, PSAD, (f/t)/PSAD, and fPSAD

**Fig. 1:**
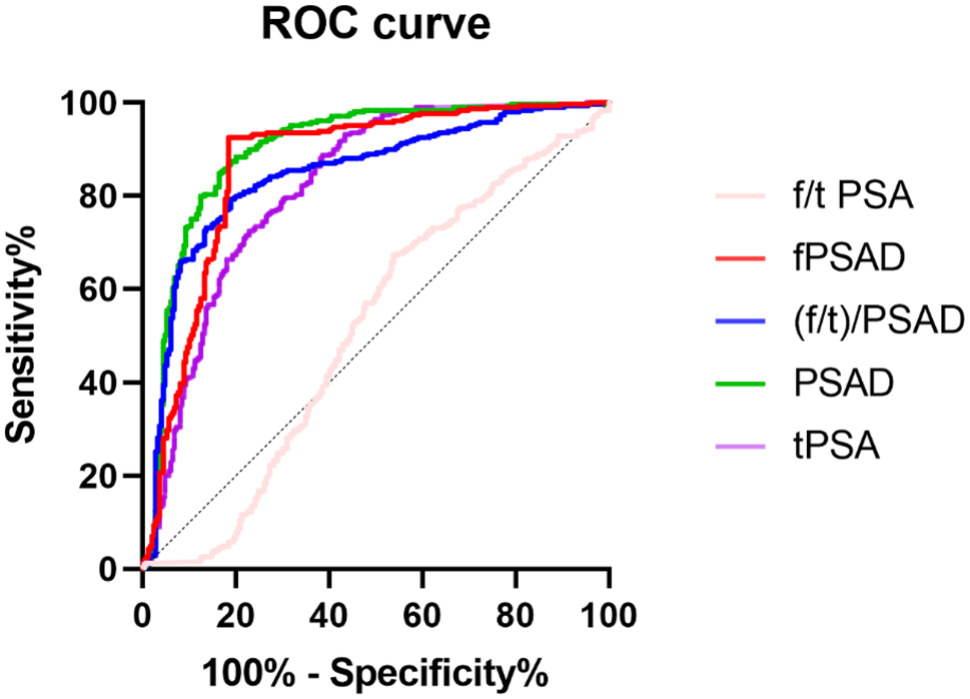
ROC curve for prostate cancer diagnosis

The ROC curves of the five parameters were merged using GraphPad Prism 9 (Fig. 1). The AUC values of tPSA, f/tPSA, PSAD, (f/t)/PSAD, and fPSAD were 0.820, 0.511, 0.900, 0.846, and 0.867 respectively (Table 2). tPSA, PSAD, (f/t)/PSAD, and fPSAD had high accuracy in predicting PCa, while f/tPSA failed to predict PCa accurately. From the sensitivity and specificity in the ROC curve, the maximum Youden index of (f/t)/PSAD and fPSAD was obtained, based on which the cutoff values were calculated as 0.29 and 0.062, respectively. The cutoff values of tPSA, f/tPSA, and PSAD were 4 ng/mL, 0.16, and 0.15, respectively. Then, the sensitivity, specificity, Youden index, and concordance of each indicator were calculated according to the above cutoff values. The results showed that fPSAD was inferior to tPSA, f/tPSA, (f/t)/PSAD, and PSAD in the diagnostic sensitivity for PCa but its specificity and concordance were significantly higher than those of the latter two, which indicates that fPSAD has the highest accuracy in the diagnosis of PCa.

**Table 2:** Comparison of diagnostic performance of each indicator for prostate cancer

	AUC	Cut-off	Yi	Sensitivity	Specificity	Concordance	Kappa	*p*
tPSA	0.820	4.00	0.059	96.80%	9.10%	48.21%	0.053	0.005
f/tPSA	0.511	0.16	0.029	58.60%	44.30%	50.72%	0.029	0.481
PSAD	0.900	0.15	0.280	96.00%	32.00%	60.57%	0.260	0.000
(f/t)/PSAD	0.846	0.29	0.604	81.10%	79.30%	80.11%	0.600	0.000
fPSAD	0.867	0.06	0.734	81.50%	91.90%	87.28%	0.740	0.000

tPSA PSAD (f/t)/PSAD and fPSAD have high accuracy in predicting prostate cancer while f/tPSA cannot consistently predict malignant tumors. tPSA: total PSA, fPSA: free PSA, f/t PSA = fPSA/tPSA, PSAD: prostate-specific antigen density = PSA/PV, (f/t)/PSAD = f/tPSA/PSAD, fPSAD: free prostate-specific antigen density = fPSA/PV

### Diagnostic performance in predicting PCa based on stratification

**Fig. 2:**
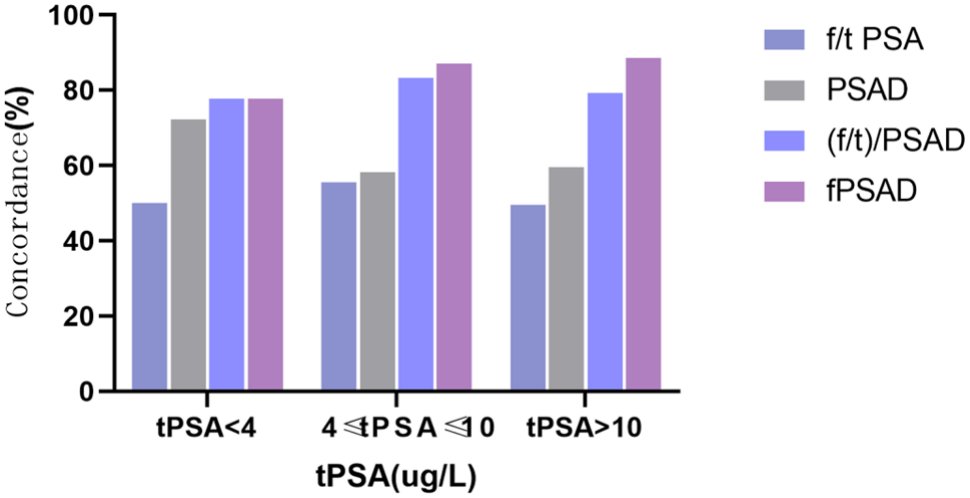
Stratification by PSA

Regardless of the PSA level (> 10 ng/mL, < 4 ng/mL, or 4–10 ng/mL), the specificity and concordance of fPSAD were far superior despite its slightly poor sensitivity (Table 3). The results suggested that fPSAD has the highest accuracy in predicting PCa regardless of the PSA level. The concordance of the indices is compared in Fig. 2.

**Table 3:** Diagnostic performance in predicting prostate cancer based on different PSA levels

	Sensitivity	Specificity	Concordance
tPSA < 4 ng/mL			
f/tPSA	12.50%	60.70%	50.00%
PSAD	0.00%	92.90%	72.22%
(f/t)/PSAD	0.00%	100.00%	77.78%
fPSAD	0.00%	100.00%	77.78%
4 ≤ tPSA ≤ 10 ng/mL			
f/tPSA	93.30%	49.50%	55.56%
PSAD	93.30%	52.70%	58.33%
(f/t)/PSAD	33.30%	91.40%	83.33%
fPSAD	13.30%	98.90%	87.04%
tPSA > 10 ng/mL			
f/tPSA	57.70%	39.60%	49.50%
PSAD	99.50%	11.50%	59.90%
(f/t)/PSAD	86.90%	69.80%	79.21%
fPSAD	89.20%	87.90%	88.61%

**Fig. 3:**
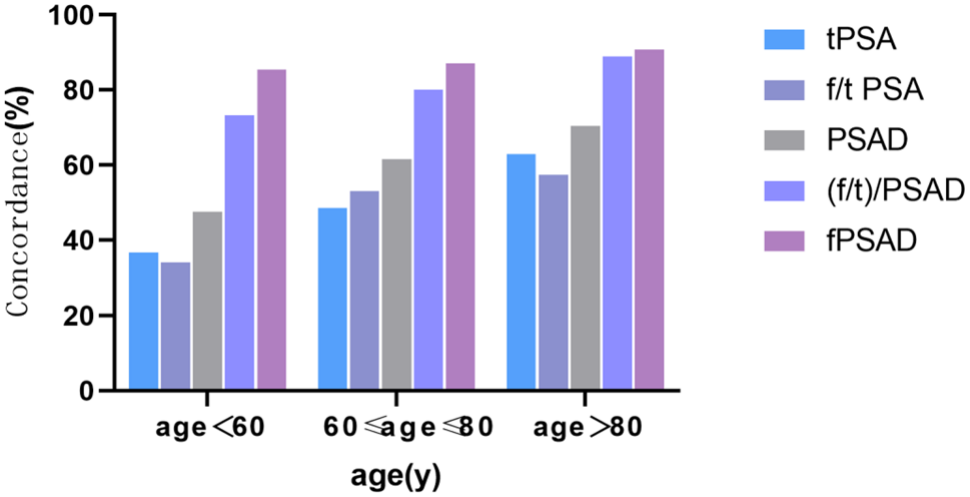
Stratification by age

Regardless of the age group (> 80 year, < 60 year, or 60–80 years), the concordance of fPSAD was significantly higher than that of other indicators (Table 4). The results suggest that fPSAD has the highest accuracy in predicting PCa in the young, middle-aged, and elderly populations. The comparison results of concordance are shown in Fig. 3.

**Table 4:** Diagnostic performance in predicting prostate cancer based on different ages

	Sensitivity	Specificity	Concordance
Age < 60 y			
tPSA	95.20%	16.40%	36.59%
f/tPSA	52.40%	27.90%	34.15%
PSAD	95.20%	31.10%	47.56%
(f/t)/PSAD	81.10%	70.50%	73.17%
fPSAD	71.40%	90.20%	85.37%
60 y ≤ age ≤ 80 y			
tPSA	96.30%	7.30%	48.66%
f/tPSA	60.20%	46.80%	53.04%
PSAD	95.30%	32.30%	61.56%
(f/t)/PSAD	78.50%	81.40%	80.05%
fPSAD	79.60%	93.60%	87.10%
Age > 80 y			
tPSA	100.00%	4.80%	62.96%
f/tPSA	51.50%	66.70%	57.41%
PSAD	100.00%	23.80%	70.37%
(f/t)/PSAD	93.90%	81.00%	88.89%
fPSAD	97.00%	81.00%	90.74%

**Fig. 4:**
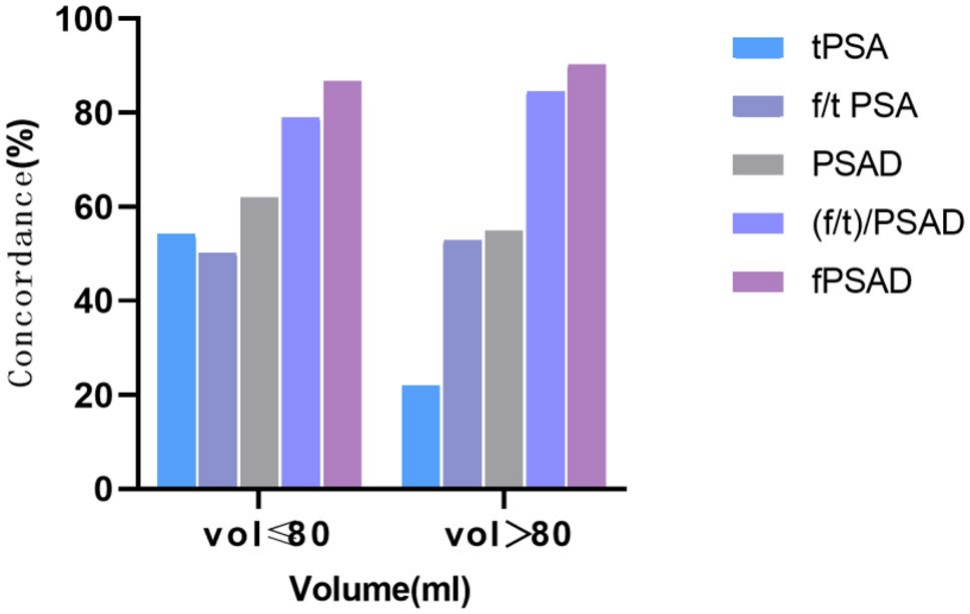
Stratification by prostate volume

The concordance of fPSAD was significantly higher than that of other indicators regardless of the PV level (≤ 80 mL or > 80 mL) (Table 5). These results indicate that fPSAD has the highest accuracy in predicting PCa in large or small prostate patients. The concordance is compared in Fig. 4.

**Table 5:** Diagnostic performance in predicting prostate cancer based on different prostate volumes

	Sensitivity	Specificity	Concordance
PV ≤ 80 mL			
tPSA	96.50%	11.10%	54.19%
f/tPSA	60.30%	40.00%	50.22%
PSAD	96.10%	27.10%	61.89%
(f/t)/PSAD	83.40%	74.70%	79.07%
fPSAD	80.80%	92.90%	86.78%
PV > 80 mL			
tPSA	100.00%	3.60%	22.12%
f/tPSA	40.00%	56.00%	52.88%
PSAD	95.00%	45.20%	54.81%
(f/t)/PSAD	55.00%	91.70%	84.61%
fPSAD	90.00%	90.50%	90.38%

## Discussion

In China, PSA is the most commonly used parameter for PCa screening, characterized by high sensitivity but low specificity. In order to find parameters that can accurately predict PCa several PSA-derived parameters have emerged in clinical practice, such as f/tPSA, (f/t)/PSAD, PSAD, PSAV, p2PSA, and Prostate Health Index (PHI), but they are difficult to widely popularize and apply in clinical practice due to unsatisfactory diagnostic efficiency [10] or high testing price [11, 12]. Therefore, we wondered if there was an indicator that was both more efficient in diagnosing PCa and clinically accessible. Research has found that fPSAD was of high clinical value [9], as it had a high concordance and could be calculated only from fPSA and PV, giving it great potential for clinical popularization and application. In evaluating the diagnostic value of fPSAD, this study verified the high concordance of fPSAD.

As guidelines recommended, the PSA threshold triggering prostate biopsy is 4 ng/mL. However, since the PSA value is susceptible to alteration inflammation, ejaculation, and anal digital examination, it has some limitations in the diagnosis of PCa so the elevation in PSA does not necessarily indicate PCa. Considering the higher median PSA level in newly diagnosed PCa patients in China than in Western countries [13], some scholars also suggest that the “diagnostic gray zone” of PCa be broadened to 4–20.0 ng/mL in order to reduce unnecessary biopsy [14]. Therefore, finding an indicator that can effectively predict PCa under different PSA levels is essential. To test whether fPSAD has the above advantages, this study further stratified the PSA levels. The results revealed that regardless of PSA in the gray zone or other ranges, fPSAD had the highest accuracy in predicting PCa with stable and reliable efficiency. More importantly, an unnecessary prostate biopsy can be reduced due to the high specificity of fPSAD.

Some scholars have found that age and PV affect the diagnosis of PCa [15–18]. It is considered that age is related to the diagnosis and prognosis of PCa [19] and serves as an essential factor causing an increased risk of PCa [20, 21]. Shan et al. [22] found that these screening indicators have different diagnostic values in different age groups. Given the correlation between age and PCa, it is necessary to compare the diagnostic value of parameters for PCa screening in different age groups. Some studies have found that PV is an independent risk factor for PCa [23], and PV may affect the predictive accuracy of PSA [24]. Liu et al. [25] argued that PV in patients with PCa is smaller than in patients with BPH, and a larger PV corresponds to a lower positive biopsy rate. Further stratified evaluation is necessary for men with a significantly increased PV (> 80 mL) [26] vs. a small PV. The results of this study showed that fPSAD as a new clinical indicator, had higher diagnostic values for PCa in different age groups and different PV groups, suggesting that fPSAD can be used as an independent and reliable predictive indicator in clinical practice.

In the last few years, more biomarkers, including serum, urine, and tissue biomarkers, have emerged to improve the detection before prostate biopsy [27, 28]. However, most markers are expensive to measure, restricting their use. The parameters in this study have certain advantages over those in previous studies. For example, fPSAD is easily accessible without additional examinations, so it is cheap and ready to popularize in hospitals at all levels. Moreover, fPSAD has higher concordance than traditional parameters such as PSAD and (f/t)/PSAD. To better manage patients with abnormal PSA, fPSAD, imaging examination results, and other related data can be analyzed to rationally screen out those who do not need prostate biopsy temporarily, thereby reducing unnecessary prostate biopsy. In addition, fPSAD can be combined with PI-RADS into an evaluation system and a risk prediction model. fPSAD can be incorporated into the Prostate Cancer Prevention Trial Risk Calculator (PCPT-RC) and the European Randomized Study of Screening for Prostate Cancer Risk Calculator (ERSPC-RC) to improve the prediction of aggressive PCa. However, the success of this approach remains to be further studied.

There are some limitations to this paper. First, this single-center study had a particular selection bias and an insufficient sample size. The small sample size in stratified analysis affected the sensitivity, but the concordance remained stable. Second, only some cases had pathological results of radical prostatectomy or plasma kinetic resection of the prostate, whereas most cases had pathological results of prostate biopsy with a 12 + X needle. Although all patients were followed up for 3–6 months, the missed diagnosis was still possible. In addition, the *Chinese Guidelines for the Diagnosis and Treatment of Urological Diseases* state that men with PSA = 4–10 ng/mL require biopsy only when f/tPSA < 0.16 or PSAD > 0.15, which could have biased the results. Since this was a retrospective study, the predictive accuracy of these parameters for PCa remains to be compared by prospective studies.

## Conclusions

fPSAD has a higher diagnostic value for PCa than tPSA, f/tPSA, (f/t)/PSAD, and PSAD, with higher specificity and concordance. With the optimal cutoff value of 0.062, this new indicator can well predict the risk of PCa significantly improve the clinical diagnostic rate of PCa and reduce unnecessary biopsy.

## Data Availability

The dataset generated or analyzed during the current study are not publicly available due to identifiable patient information but are available from the corresponding author upon reasonable request.
